# Antioxidant Properties of *cis*-*Z*,*Z'*-3a.7a',7a.3a'-Dihydroxy- ligustilide on Human Umbilical Vein Endothelial Cells *in Vitro*

**DOI:** 10.3390/molecules18010520

**Published:** 2013-01-02

**Authors:** Weidong Li, Yu Wu, Xuedong Liu, Cuiping Yan, Dan Liu, Yang Pan, Guangming Yang, Fangzhou Yin, Zebin Weng, Ding Zhao, Zhipeng Chen, Baochang Cai

**Affiliations:** 1 College of Pharmacy, Nanjing University of Chinese Medicine, Nanjing 210046, China; 2 Engineering Center of State Ministry of Education for Standardization of Chinese Medicine Processing, Nanjing University of Chinese Medicine, Nanjing 210046, China; 3 College of Pharmacy, China Pharmaceutical University, Nanjing 210009, China; 4 Department of R&D, Nanjing Haichang Chinese Medicine Group Corporation, Nanjing 210061, China

**Keywords:** *cis*-*Z*,*Z'*-3a.7a',7a.3a'-dihydroxyligustilide, antioxidant, human umbilical vein endothelial cell, cell cycle

## Abstract

A new chemical component, *cis*-*Z,Z'*-3a.7a',7a.3a'-dihydroxyligustilide, was isolated from *Angelica sinensis* and its structure elucidated from its NMR and MS spectra and confirmed by X-ray single crystal diffraction analysis. We also explored the antioxidative properties of *cis*-*Z,Z'*-3a.7a',7a.3a'-dihydroxyligustilide on human umbilical vein endothelial cells (HUVECs) against injuries induced by hydrogen peroxide (H_2_O_2_) using an MTT assay and flow cytometry analysis. In addition, the activities of superoxide dismutase (SOD), malondialdehyde (MDA), lactate dehydrogenase (LDH), nitric oxide (NO) and reactive oxygen species (ROS) were determined. We found that *cis*-*Z,Z'*-3a.7a',7a.3a'-dihydroxyligustilide increased the viability of HUVECs injured by H_2_O_2_ in a dose-dependent manner, reduced the apoptosis of HUVEC, and enhanced HUVEC proliferation. Our results demonstrated the remarkable *in vitro* antioxidative activities of this compound, indicating that it could be a potential antioxidant with protective effects against H_2_O_2_-induced HUVEC injuries.

## 1. Introduction

Radix *Angelicae Sinensis* (AS) (Oliv.) Diels (Umbelliferae), also known as Dang-gui, is one of the most important Traditional Chinese Medicines (TCMs). TCM theory holds that atherosclerosis results from blood stasis, which is usually caused by stagnant blood and poor blood flow. AS is one of the TCMs commonly used in the clinic to improve blood circulation and remove blood stasis. In the clinical context, AS is mainly used for the treatment of anemia, gynecological diseases, strokes, hypertension and coronary heart embolism [1]. As we know, several chronic human diseases are thought to be associated with oxidative damage, including diabetes mellitus, cardiovascular diseases, cancer, atherosclerosis, neurodegenerative diseases, and oxidative stress is also believed to be a major factor associated with aging [2,3,4]. According to the clinical role of AS, it is suggested that AS plays an important role as an antioxidant. AS contains of a variety of constituents, such as essential oils, aromatic compounds, coumarins, terpenes, polyynes, organic acids, polysaccharides, vitamins, amino acids and other components [5]. The main bioactive components of AS include ligustilide, ferulic acid (FA), and polysaccharides. However, previous studies have mainly focused on the antioxidant activities of the polysaccharides [6] and FA [7], and so for, little is known with respect to the antioxidant activity of ligustilide. It has been reported that *Z*-ligustilide isolated from AS protected against hydrogen peroxide (H_2_O_2_)-induced cytotoxicity in PC12 cells and forebrain I/R by enhancing antioxidant defenses [8]. Because of the instability of ligustilide, studies in-depth and detailed investigations into this compound are needed. Supercritical fluid extraction (SFE) possesses several advantages over traditional liquid-solvent-based extraction methods and has been extensively studied for separation of many bioactive compounds [9], so we used SFE technology to separate ligustilide from AS.

Reactive oxygen species (ROS) are highly reactive metabolites of oxygen, such as superoxide (O_2_·^−^), hydrogen peroxide (H_2_O_2_), and peroxynitrite (ONOO^−^). ROS can change cellular signaling systems and cause damage to lipids, proteins and DNA [10], resulting in oxidative stress. Enhanced activity of oxidant enzymes and/or reduced activity of antioxidant enzymes cause oxidative stress [11], which is characterized by increased endogenous production of ROS such as superoxide anion and H_2_O_2_. ROS can react with and damage cellular components, but ROS can also be used in signal transduction [12]. Because H_2_O_2_ does not have an unpaired electron it is less reactive than other ROS. It has been found that H_2_O_2_ can cause endothelial cell injury by inducing mitochondrial dysfunction [13,14]. Mitochondrial permeability transition is a mechanism causing mitochondrial failure. Mitochondrial permeability transition can cause necrosis due to ATP depletion [15]. The vascular endothelial system as the largest endocrine organ in the human body plays an important role in maintaining body homeostasis. Human umbilical vein endothelial cells (HCAECs) are significant for the elucidation of the antioxidant effects of AS. The aim of this study was to separate ligustilide from AS and detect its antioxidant activity on the *in vitro* oxidative-stress-mediated injury of HCAECs. In the present study, we directly isolated cis-*Z,Z'*-3a.7a',7a.3a'-dihydroxyligustilide from AS for the first time and aimed at investigating its effect on oxidative stress-mediated injury of human umbilical vein endothelial cells (HUVECs) induced by H_2_O_2_ *in vitro*.

## 2. Results and Discussion

### 2.1. Results

#### 2.1.1. Structure Elucidation

**Figure 1:**
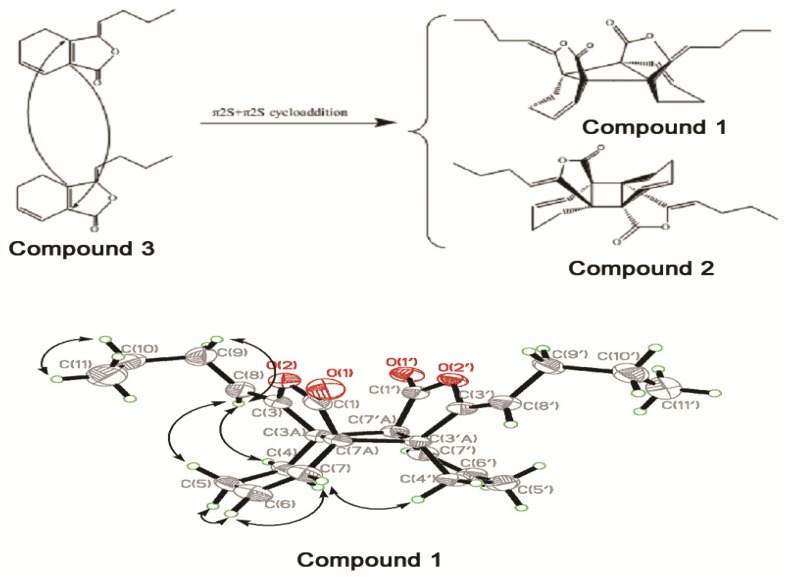
Postulated biosynthetic pathways of compounds **1** and **2**, X-ray crystal structure and key NOESY correlations of compound **1**.

Three compounds were isolated from AS and purified. Elucidation and identification of compound structure were performed with ^1^H-NMR, ^13^C-NMR, ^1^H,^1^H-COSY, NOESY, HMBC and X-ray single crystal diffraction analyses. Compound **1** was isolated as colorless crystals. The formula was determined as C_24_H_28_O_4_ by HR-ESI-MS (*m/z* 403.1891 [M+Na]^+^ calcd. 403.1883) with eleven degrees of unsaturation. The IR spectrum of compound **1** indicated the presence of lactone (1,780, 1,040 cm^−1^) and cyclohexene groups (1,704 cm^−1^). The ^1^H-NMR and ^13^C-NMR analyses showed only 14 hydrogen atoms and 12 carbon atoms, suggesting that compound **1** was an isometric dimeric compound. The ^1^H-NMR and ^13^C-NMR spectra of compound **1** resembled those of the known compound *trans*-*Z*,*Z'*-3a.7'a,7a.3'a-diligustilide (**2**) [16], which was reasonably ascribed by the COSY, HMQC and HMBC (Figure 1). The structure of compound **1** was determined by the observed cross-peak between H-7 and H-4'a in the NOESY spectrum. Moreover, the structure and stereochemistry of compound **1** were confirmed by X-ray diffraction [17]. As shown in Figure 1 and Table 1, the two lactone groups of compound **1** were in a *cis*-configuration, while in compound **2** they were in a *trans*-configuration. The deshielding effect of the carbonyl resulted in the chemical shift of C-3a (δ 51.7), C-7a (δ 50.4), C-6 (δ 134.1) being resonated downfield by 2.0, 1.0, 1.9 ppm, respectively. Similarly, the shielding effect of the carbonyl made the chemical shift of C-1, C-3, C-4 resonate upfield by 0.9, 1.3 and 2.5 ppm, respectively, compared to compound **2**. Therefore, compound **1** was elucidated as *endo*-*Z*,*Z'*-3a.7'a,7a.3'a-diligustilide. NMR data of compound **3** were consistent with previous reports [18,19]. H-8 (δ 5.22) determined the compound of the Z-type. Therefore, compound **3** was elucidated as *Z*-ligustilide.

**Table 1:** ^1^H-NMR (500 MHz) and ^13^C-NMR (125 MHz) spectral data of compound **1** (in CDCl_3_) (δ, ppm; *J*, Hz).

No.		δ_C_ (DEPT)	δ_H_ *J* (Hz)	HMBC(H→C) *	NOESY
1	1'	172.7 (s)			
3	3'	148.9 (s)			
3a	3'a	51.7 (s)			
4	4'	24.6 (t)	1.92 (α-H, m); 1.85 (β-H, m)	C-3, 3a, 5, 6, 7, 7a	H-7, H-8
5	5'	20.7 (t)	2.13 (α-H, m); 1.92 (β-H, m)	C-6, 7, 7a	H-6, H-8
6	6'	134.1 (d)	6.19 (1H, ddd, 10.0, 6.5, 1.0)	C-1, 3a, 4, 5, 7	H-5, H-7
7	7'	120.9 (d)	5.80 (1H, dd, 10.0, 1.0)	C-1, 5, 7a,	H-4', α, H-6
7a	7'a	50.4 (s)			
8	8'	107.8 (d)	4.85 (1H, t, 7.5)	C-3, 7, 7a, 10	H-4, H-5, H-9
9	9'	27.4 (t)	2.20 (α-H, m); 2.13 (β-H, m)	C-3, 8	H-8, H-10
10	10'	22.5 (t)	1.42 (2H, m)	C-8, 9, 11	H-9, H-11
11	11'	13.7 (q)	0.91 (3H, t, 7.5)	C-9, 10	H-10

* As an isometric dimeric compound, the long-range coupling correlations of the half part of the molecular structure was presented here, the another part was exactly the same.

#### 2.1.2. Antioxidant Activity

**Figure 2:**
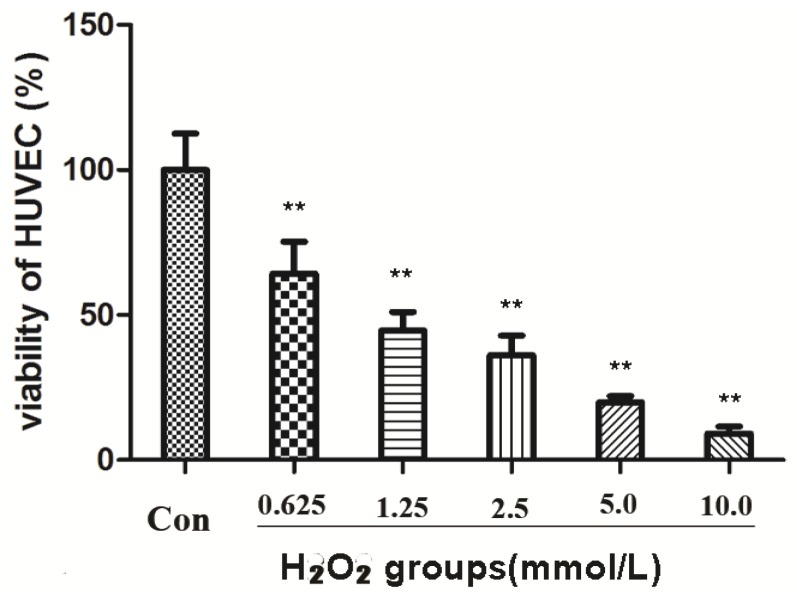
Effects of H_2_O_2_ on viability of HUVECs (** *p* < 0.01 *vs.* control).

**Figure 3:**
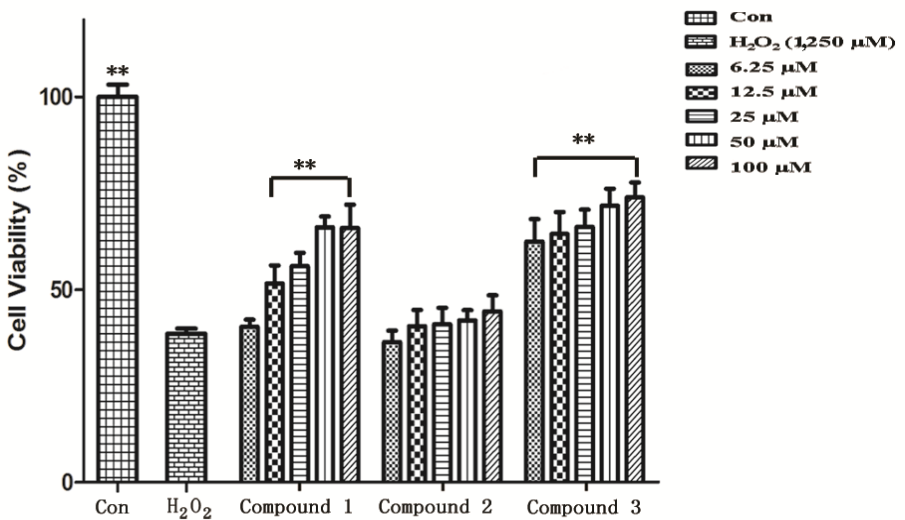
The protective effect of compounds **1** and **2** on H_2_O_2_-induced cytotoxicity in HUVECs. HUVECs were incubated with compounds **1** and **2** at the indicated concentrations for 24 h, and then 1,250 μmol/L H_2_O_2_ was added for an additional stimulation for 2 h. Cell viability was examined by MTT assay. The data were expressed as mean ± SD (n = 6). ** *p* < 0.01 *vs.* H_2_O_2_ treatment.

HUVECs at the mid-log phase were incubated with H_2_O_2_ at 0.625, 1.25, 2.5, 5.0, and 10.0 mM for 2 h. The survival rate dropped to 45% at 1.25 mM concentration of H_2_O_2_ compared to the control group (** *p* < 0.01) (Figure 2). To ensure moderate cell damage, 1.25 mM H_2_O_2_ was used in all experiments as model group to induce oxidative stress in HUVECs. MTT assay showed that compounds **1** and **3** exerted a potent anti-oxidative effect in a dose-dependent manner (Figure 3). Treatment with compound **1** (12.5 μM) made the survival rate of HUVECs increase to 51.22%, while only 38.56% cell survival was observed in the model group. When the dose of compound **1** reached 50 μM, the survival rate increased to 65.81% compared to the model group. Compared to compound **3**, the effect against oxidative stress on HUVEC cells was considerable (65.81 ± 2.71 *vs.* 71.40 ± 4.42, 50 μM dosage). Both compounds showed significant differences compared to the model group (** *p* < 0.01). However, compound **2** had no significant protective effect against oxidative damage in HUVECs (*p* > 0.05). The IC_50_ values of compound **1** and compound **3** were 15.14 and 0.55 µM, respectively. However, The IC_50_ value of compound **2** greater than 100.00 µM.

**Figure 4:**
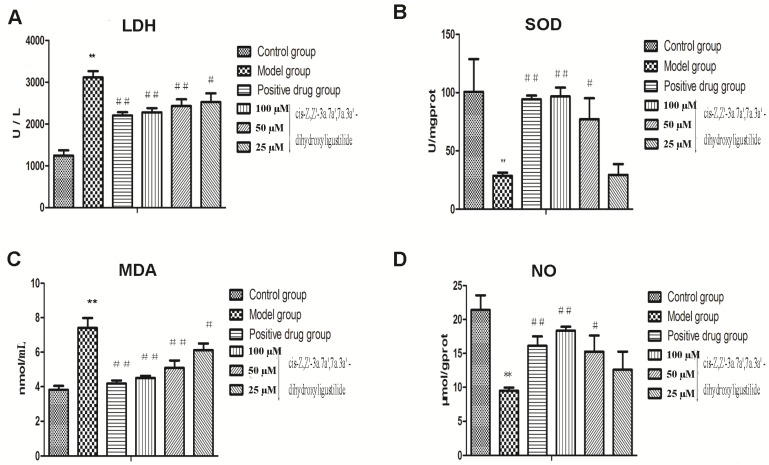
Effects of compound **1** on LDH, SOD, MDA and NO contents in HUVECs injured by H_2_O_2_. Cells were treated with compounds 1 at indicated concentrations for 24 h, and Vitamin E (0.001 µM) as a positive drug. The levels of LDH and MDA were decreased, while SOD and NO were increased compared to the model group (** *p* <0.01 *vs.* control, ## *p* <0.01, # *p* < 0.05 *vs.* model).

Compound **1** as a new compound isolated directly from AS showed potent anti-oxidative activity in the preliminary test and it was worthy of further research. Vitamin E was used in our experiments as a positive control and we determined the contents of LDH, SOD, MDA and NO (Figure 4). Our results demonstrated that there was a marked increase in LDH leakage in the model group compared to the control group (3,115.19 ± 150.98 *vs.* 1,243.63 ± 129.21 U/L, ** *p* < 0.01). However, LDH leakage was significantly reduced to 2524, 2,433 and 2,277 U/L, respectively, by treatment with 25, 50 and 100 μM of compound **1** compared to the model group. Vitamin E (0.001 µM) as a positive drug also decreased the LDH leakage (2,205.91 ± 80.35 U/L). Incubation with H_2_O_2_ led to significant decrease in the intracellular SOD activity from 100.68 U/mgprot to 28.50 U/mgprot (** *p* < 0.01), and the intracellular MDA production was significantly increased from 3.82 nmol/mL to 7.40 nmol/mL compared to the control group (** *p* < 0.01). Treatment with compound **1** at the indicated doses (25, 50 and 100 µM) increased the SOD activity to 29.43, 77.14 and 96.66 U/mgprot, respectively, and decreased the MDA level to 6.11, 5.09 and 4.50 nmol/mL, respectively, compared to the model group. In the evaluation of SOD activity, except the low-dose group, other groups had a significant difference compared to the model group and in the assessment of MDA level, all groups had a significant difference compared to the model group. Furthermore, inhibitory effect of H_2_O_2_ on NO production was observed, because NO release was decreased from 21.40 µmol/gprot to 9.50 µmol/gprot compared to the control group (** *p* < 0.01). Treatment with compound **1** at moderate and high doses increased NO leveld to 15.25 and 18.35 μmol/gprot, respectively, with significant differences compared to the model group. The mean fluorescence intensity of ROS decresed significantly from 149.7 to 80.05 compared with model group.

**Figure 5:**
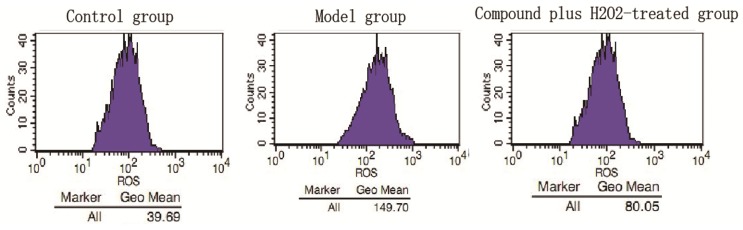
Effects of compound **1** on the ROS contents in HUVECs injured by H_2_O_2_. Cells were treated with compounds **1** (50 µM) for 24 h then measured using an DCFH-DA probe according to the manufacturer’s instructions. The level of ROS was increased in the model group (H_2_O_2_-treated group), while treatment with compound **1**, the content of ROS was decreased significantly.

Figure 5 shows the effect of compound **1** on HCAEC cultures subjected to ROS injury. Compound **1** (50 μM) significantly decreased H_2_O_2_-induced ROS injury. Furthermore, flow cytometry analysis showed that exposure of HUVECs to H_2_O_2_ resulted in a significant increase in the G2 phase accompanied by decreased distribution in the S phase. 

**Figure 6:**
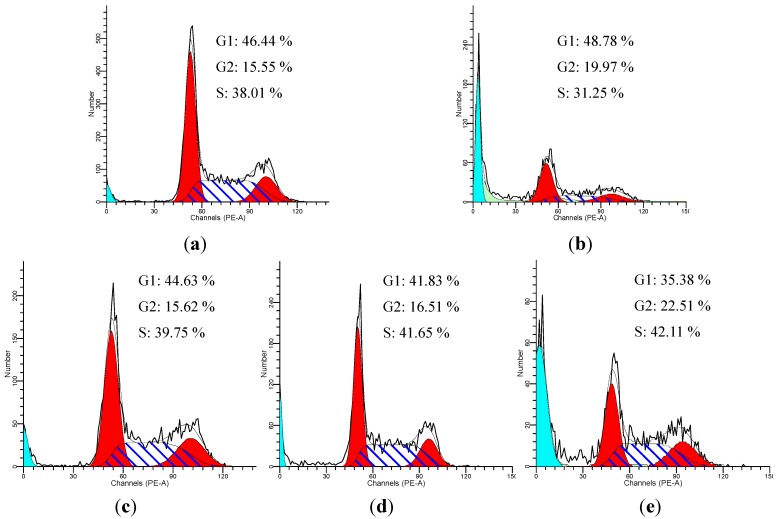
*cis*-*Z,Z'*-3a.7a',7a.3 a'-Dihydroxyligustilide effectively relieves H_2_O_2_-induced cell cycle arrest. Treatment with compound **1** led to a decrease in G2 phase and increase in S phase. (**a**) control cells; (**b**) model cells; (**c**–**e**): cells treated with 100, 50, 25 μmol/L of endo-*Z,Z'*-3a.7a',7a.3a'-diligustilide, respectively.

Cells showed G2/M arrest from 15.55% in the control group to 19.97% in the model group and showed apoptosis peak. Treatment with compound **1** (*cis*-*Z,Z'*-3a.7a',7a.3 a'-dihydroxyligustilide) at 25, 50 and 100 μM for 24 h resulted in 22.51%, 16.51% and 16.12% of cells in the G2/M phase (Figure 6). Treated with compound **1** can significantly reduce the G2/M arrest.

### 2.2. Discussion

Compounds **1** and **2** were dimeric derivatives of two ligustilide monomers formed via cycloaddition reactions as postulated in Figure 1. Compounds **1** and **2** were probably generated by an alternative cycloaddition mechanism that formed the cyclobutane ring [20]. In addition to the two phthalide dimers reported in this paper, monomer units and linkage of structural skeletons of 15 phthalide dimers from AS were summarized in Table 2 [21,22,23,24,25,26,27,28]. One of the compounds was extracted directly from AS for the first time and we evaluated its antioxidant activity in H_2_O_2_-injuried HUVECs.

It is well-known that *Z*-ligustilide is one of the main components of the essential oil of AS, and it was reported to protect against H_2_O_2_-induced injury in PC12 cells [5]. We compared the anti-oxidative activity of three obtained compounds by MTT assay. We used H_2_O_2_ as an exogenous generating system of free radicals to establish oxidative stress model. According to the MTT experiments, it could be postulated that *Z*-ligustilide exhibited the strongest antioxidant activity among the three compounds, which was closely related to the unbroken conjugated system in its structure. The difference of stereoscopic structure between compounds **1** and **2** probably led to their different antioxidant activities. Compound **1** showed good anti-oxidative activity in the preliminary test and it was worthy of further research.

**Table 2:** Overview of known phthalide dimers, which have been isolated from *A. sinensis*.

Number	Name	Monomer unit(s)	Linkage	References
1	*Z,Z'*-3.3'a,7.7'a-diligustilide	*Z*-ligustilide	3a-7′a, 7a-3′a	[16]
2	Angelicide	*Z*-ligustilide	6-8′, 7-3′	[21]
3	Gelispirolide	*Z*-ligustilide and butylidenephthalide	6-8′, 7-3′	[22]
4	Riligustilide	*Z*-ligustilide	6-8′, 7-3′	[22]
	Levistolide A	*Z*-ligustilide	6-6′, 7-3′a	
5	Levistolide B	*Z*-ligustilide and *E*-ligustilide	6-6′, 7-3′a	[23]
6	*Z*-3′,8′,3′a,7′a-tetrahydro-6,3′,7,7′a-diligustilide-8′-one	*Z*-ligustilide	6-3′, 7-7′a	[23]
7	*Z,Z′-*3.3′,8.8′-diligustilide	*Z*-ligustilide	3-3′, 8-8′	[22]
8	*E*,*E′*-3.3′,8.8′-diligustilide	*E*-ligustilide	3-3′, 8-8′	[24]
9	Senkyunolide O	*Z*-ligustilide and *E*-ligustilide	6-6′, 7-3′a	[24]
10	Tokinolide B	*Z*-ligustilide	3-3a′, 8-6′	[24]
11	E-232	*Z*-ligustilide and *E*-ligustilide	3a-8′, 6-3′	[25]
12	Sinaspirolide	*Z*-ligustilide and butylidenephthalide	3-3′a, 8-7′a	[26]
13	Ansaspirolide	*Z*-ligustilide and butylidenephthalide	3-3′a, 8-6′	[27]
14	3-3′ *Z*,-6.7′,7.6′-diligustilide	*Z*-ligustilide	6.7′, 7.6′	[28]

Vitamin E has receives considerable attention for its importance as an antioxidant which acts to protect the cells from the damage of free radicals [29], so we choose vitamin E as a positive control in our experiments. Enzymatic sources of ROS are the mitochondrial electron transport chain, lipooxygenase, cyclooxygenase, cytochrome P450s, xanthine oxidase, NAD(P)H oxidase, uncoupled eNOS, and other hemoproteins. These systems produce a 1-electron reduction of molecular oxygen to form a superoxide anion. Superoxide anion converts to H_2_O_2_ either spontaneously or by the SOD enzyme pathway. Subsequently H_2_O_2_ converts to water and oxygen through catalase, glutathione peroxidase, and thiols. H_2_O_2_ has three fates: it reacts with nitric oxide to form nitrogen dioxide anion; it is catalyzed by enzymes such as glutathione peroxidase and catalase to form water and oxygen; or, in the presence of heavy metals, it undergoes a Fenton reaction to form a hydroxyl radical [30]. It is known that LDH release into the media was used as an indicator of the integrity of cell membranes or necrosis in response to the oxidant burden [31]. SOD is a paramount antioxidant enzyme to defense the body against superoxide free radicals. In brief, SOD keeps oxygen under control. MDA is a breakdown product of the oxidative degradation of cell membrane lipids and generally considered an indicator of lipid peroxidation and also indirectly reflects cellular damage. NO is important in maintaining vasodilatation. NO is produced by oxidation of L-arginine catalyzed by NO synthases (NOS). NO toxicity is mainly mediated by peroxinitrite, and its reaction product (ONOO^−^) is an attractive candidate for cytotoxicity [32,33]. We detected various factors (SOD, MDA, LDH, NO and ROS) to comprehensive evaluate antioxidant activity of compound **1**. As we know, mitochondria are the major source of ROS production and often targets of high ROS exposure with deleterious consequences, such as oxidative damage to mitochondrial DNA. Mitochondrially generated H_2_O_2_ can also act as a signaling molecule in the cytosol, affecting multiple networks that control, for example, cell cycle, stress response, energy metabolism, and redox balance [34,35,36]. Interestingly, the ability of H_2_O_2_ to promote apoptosis, through the activation of JNK [37] and NF-kB [38]. ROS activation of JNK can induce extrinsic or intrinsic apoptotic signaling. TNF-α is a potent activator of the MAPK cascade, and TNF-α induced ROS cause oxidation and inhibition of JNK-inactivating phosphatases, which is required for cytochrome c release and caspase 3 cleavage, as well as necrotic cell death [39].

In this study we showed that compound **1** has a protective effect on HCAECs facing oxidative -stress- induced cell injury in dose-dependent manner. We used 50 μM compound **1** as the middle concentration, and we found that the survival rate increased to 65.81% and IC_50_ values of compound **1 **was 15.14 µM. LDH, SOD, MDA and NO contents of the compound **1** group were adjusted towards the control group. Our data showed that 50 μM compound **1** decreased ROS generation, suggesting that the ROS- scavenging capacity of compound **1** may be related to decreased cell death. Cells showed G2/M arrest in the model group compared, however treated with compound **1** can significantly reduce the G2/M arrest. The percentage of G2 phase decreased and the percentage of S phase increased. Its anti-oxidative activity could effectively relieve the H_2_O_2_-induced cell cycle arrest in HUVECs beneficial to the resumption of cell proliferation. Our results demonstrated remarkable antioxidative activities of this compound *in vitro*, indicating that this compound could be a potential antioxidant with protective effects on HUVEC injuries induced by H_2_O_2_.

## 3. Experimental

### 3.1. General Experimental Procedures

Silica gel for column chromatography (200–300 mesh) and thin layer chromatography (TLC) plates (10–40 μm) were the products of Qingdao Haiyang Chemical (Qingdao, China). IR spectra were taken on a MPA FT-IR spectrometer in KBr discs. NMR spectra were measured on a Bruker AV-500 MHz (500 MHz for ^1^H-NMR and 125 MHz for ^13^C-NMR) spectrometer (Bruker Instruments Co., Ltd., saarbrücken, Germany). HRESI-MS spectra were obtained on a Micromass Q/TOF mass spectrometer. X-ray single crystal diffraction was accomplished by Nonius CAD4 Diffractmeter. HUVECs were purchased from Biotechnology Development Co., Ltd. Nanjing KGI. (Nanjing, China). RPMI1640 medium was from Gibco (Rockville, MD, USA). Fetal bovine serum (FBS) was from Sijiqing Co., Ltd. (Hangzhou, China). Phosphate buffer saline (PBS) was from Sigma (St Louis, MO, USA). 3-(4,5-Dimethythiazol-2-yl)-2,5-diphenyltetrazolium bromide (MTT) was from Amresco Co., Ltd. (Solon, OH, USA); The light absorption was measured at 490 nm using an enzyme-linked immunosorbent assay (ELISA) reader (Bio-Rad 680, Philadelphia, PA, USA). Kits for detecting lactate dehydrogenase (LDH), superoxide dismutase (SOD), MDA and NO were purchased form Nanjing Jiancheng Bioengineering Institute (Nanjing, China). DNA content of cell detection kit was purchased from Biotechnology Development Co., Ltd. Nanjing KGI. (Nanjing, China). All solvents used were of analytical grade (Nanjing Chemical Plant, Nanjing, China).

### 3.2. Plant Materials

The dried radix of AS (8 kg) collected in September 2010 from Gansu Province of China and identified by Professor Jianwei Chen (Nanjing University of Chinese Medicine). Voucher specimens were deposited at the herbarium of Nanjing University of Chinese Medicine (Nanjing, China).

### 3.3. Extraction and Isolation

The dried radix of A.sinensis (8 kg) were cut off and extracted by supercritical fluid CO_2_ (temperature and pressure of extraction kettle, separation kettle I and II were 44, 46, 35 °C and 25, 8, 6 MPa, respectively. CO_2_ flow volume was 18 kg/h; extraction time was 4 h). The resulting extract (80 g, a brown oil) was applied to a silica gel column (4 cm × 80 cm) and eluted with petroleum ether/ethyl acetate (100:0 to 1:1, v/v) and six fractions of 250 mL each were collected. Fraction 3 was further separated using a silica gel (300–400 mesh) column (2 × 30 cm) and eluted with petroleum ether-ethyl acetate (90:1, v/v) to afford compound **2** (105 mg), and petroleum ether-EtOAc (70:1) to get compound **1** (30 mg). We performed ^1^H-NMR, ^13^C-NMR, ^1^H,^1^H-COSY, NOESY, HMBC and X-ray single crystal diffraction analyses to elucidate and confirm the structures. The extract (5.0 g) was purified by preparative TLC (1 mm, 20 cm × 20 cm) developed with *n*-hexane-acetone (7:3, v/v) and monitored under UV_254_ nm to yield compound **3** (750 mg, Rf = 0.7). Compound **1**: colorless crystals, m.p. 89–91 °C, IR (film): 1788, 1040 and 1704 cm^−1^; ^1^H-NMR and ^13^C-NMR data and HMBC, NOESY spectrum were shown in Table 1; HRESI-MS *m/z*: 403.1891 (cacld.403.1883 [M+Na]^+^). Compound **2**: colorless crystals, m.p. 85–86 °C, IR (film): 1778, 1042 and 1692 cm^−1^; ^1^H-NMR and ^13^C-NMR were shown in Table 1; HRESI-MS *m/z*: 403.1893 (cacld.403.1883 [M+Na]^+^). ^1^H-NMR (500 MHz, CDCl_3_) δ: 6.12 (1H, m, H-6/6'), 5.81(1H, dt, *J* = 10.0, 1.5 Hz, H-7/7'), 4.70 (1H, t, *J* = 7.5 Hz, H-8/8'), 2.15 (2H, m, H-9/9'), 2.12(1H, m, H-4/4'α), 1.92 (1H, m, H-4/4'β), 1.40 (2H, sext, *J* = 7.5 Hz, H-10/10'), 0.91 (3H, t, *J* = 7.5 Hz, H-11/11'). ^13^C-NMR (125 MHz, CDCl_3_; assignment by DEPT): 173.6 (C-1/1'), 150.2 (C-3/3'), 132.2 (C-6/6'), 121.7 (C-7/7'), 108.3 (C-8/8'), 49.7 (C-3a/3'a), 49.4 (C-7a/7'a), 27.4 (C-9/9'), 27.1 (C-4/4'), 22.6 (C-10/10'), 20.9 (C-5/5'), 13.6 (C-11/11'). Compound **3**: yellow oil, UV λ_max_ (MeOH): 213, 282, 329 nm. ^1^H-NMR (500 MHz, CDCl_3_) δ: 6.28 (1H, dt, *J* = 9.6, 4.3 Hz, H-7), 6.00 (1H, dt, *J* = 9.6, 4.3 Hz, H-6), 5.22 (1H, t, *J* = 7.8 Hz, H-8), 2.60 (2H, m, H-4), 2.49 (2H, m, H-5), 2.38 (2H, m, H-9), 1.50 (2H, m, H-10), 0.95 (3H, t, *J* = 7.4 Hz, H-11). ^13^C-NMR (125 MHz, CDCl_3_) δ: 167.6 (C-1), 147.1 (C-3), 148.6 (C-3a), 129.9 (C-6), 124.0 (C-7a), 117.1 (C-7), 112.9 (C-8), 28.1 (C-9), 22.4 (C-10), 18.5 (C-4) and 13.7 (C-11).

### 3.4. Antioxidant Activity *in Vitro*

#### 3.4.1. Cell Cultures and MTT Assay

HUVECs were cultured in RPMI1640 medium supplemented with 10% FBS, 100 U/mL penicillin and 100 μg/mL streptomycin in a humidified incubator under 5% CO_2_ at 37 °C. Briefly, cells at the mid-log phase were seeded in a 96-well plate at a density of 10^4^ cells per well in 100 µL medium. Firstly, cells were pre-incubated with compounds **1**–**3** at different concentrations of 6.25, 12.5, 25, 50 and 100 μM for 24 h. RPMI1640 medium was added to the control and model groups. Then we removed the original culture medium and washed cells with PBS twice. Antioxidant activity was evaluated by using HUVEC injury model induced by H_2_O_2_. Cells were treated with H_2_O_2_ (1,250 μmol/L) for 2 h. Then, 20 μL of MTT solution (5 mg/mL) was added to each well. Following 4 h of incubation at 37 °C, the supernatants were replaced with 150 μL dimethyl sulfoxide (DMSO) to dissolve the formed crystal formazan and the absorbance of each well at 490 nm was determined using an enzyme-linked immunosorbent assay (ELISA) reader. The percentage of viable cells was calculated as the relative optical density compared to the control. The cell viability ratio was calculated based on the following formula: Viability (%) = OD values of drug-treated samples/OD values of non-treated samples × 100% [13].

#### 3.4.2. LDH Release Assay

LDH as an indicator of cell injury was detected with an assay kit according to the manufacturer’s instructions. Briefly, HUVECs were treated as described above. Vitamin E (0.001 µM) was used as a positive control. Cell medium (30 µL) was taken out for the activity analysis of extracellular LDH, which could catalyze the conversion of lactate to pyruvate, and then reacted with 2,4-dinitrophenylhydrazine to give the brownish red color in basic solution. After reaction, each sample was detected and the absorbance was read at wavelength 440 nm. The experiment was conducted in triplicate.

#### 3.4.3. SOD Activity Assay

The SOD activity was detected with an assay kit according to xanthine oxidase method. The assay used the xanthine-xanthine oxidase system to produce superoxide anions, which react with 2-(4-iodophenyl)-3-(4-nitrophenol-5-phenyltetrazolium chloride) to form a red formazan dye and the absorbance at 550 nm was determined. SOD can specific inhibit superoxide anion radical and reduce the formation of nitrite that causing reduced absorbance. HUVECs were treated as described above. Vitamin E (0.001 µM) was used as a positive drug. One unit of SOD was defined as the amount of SOD inhibiting the rate of reaction by 50% at 25 °C. The experiment was performed in triplicate.

#### 3.4.4. Lipid Peroxidation Assay

MDA is a breakdown product of the oxidative degradation of cell membrane lipids. MDA was measured as an indicator of lipid peroxidation according to the thiobarbituric acid (TBA) method with a detection kit. The method was based on the spectrophotometric measurement of the red color produced during the reaction to TBA with MDA. HUVECs were treated as described above. Vitamin E (0.001 µM) was used as a positive drug. Samples were the absorbance at 532 nm was determined.

#### 3.4.5. NO Production Assay

NO production was determined indirectly by assaying the culture supernatant for accumulated nitrite, the stable end product of NO reacted with molecular oxygen. Briefly, HUVECs were treated as described above. Vitamin E (0.001 µM) was used as a positive control. After treatment, supernatants were allowed to react with Griess reagent (1% sulfanilamide, 0.1% *N*-1-naphthylethylenediamine dihydrochloride and 2.5% phosphoric acid) at room temperature for 10 min. Nitrite products in cell supernatants were determined by measuring absorbance at 550 nm.

#### 3.4.6. ROS Production Assay

To evaluate the ROS production due to DCFH-DA (Beyotime, Shanghai, China, S0033) was used. HUVECs cells at the mid-log phase were seeded in were seeded in 6-well plates at a density of 3 × 10^5^ cells/well and were grown to 60%–70% confluency. The experiment was divided into three groups: (1) The control group: each hole added 100 μL culture medium to incubated 24 h and then added 100 μL culture medium to incubated 2 h; (2) The model group: each hole added 100 μL culture medium to incubated 24 h and then added 100 μL culture medium containing H_2_O_2_ (1,250 μM) to incubated for 2 h; (3) The compound plus H_2_O_2_-treated group: each hole added 100 μL culture medium containing compound 1 (50 μM) to incubated 24 h, then added 100 μL culture medium containing H_2_O_2_ (1,250 μM) to incubated for 2 h. Each group was repeated three times. ROS generation was determined spectrofluorometrically using the DCFH-DA probe. After treatment with H_2_O_2_, ROS formation was evaluated by flow cytometry (Becton Dickinson, San Jose, CA, USA). The ROS generation was measured using a DCFH-DA probe according to the manufacturer’s instructions.

#### 3.4.7. Flow Cytometric Analysis

HUVECs were treated as described above, and then were harvested (including attached and detached cells) and fixed with 75% alcohol at 4 °C. Distribution of cells with different DNA content was analyzed using the cellular DNA flow cytometric analysis kit (KeyGEN, Nanjing, China) according to the manufacture’s instructions. The percentage of cell cycle distribution was determined using a FACScan laser flow cytometer (Becton Dickinson). The data were analyzed using the software CELLQuest.

### 3.5. Data Analysis

Data were analyzed using SPSS 15.0 (SPSS Inc., Chicago, IL, USA). All data are expressed as mean ± SD. The data were analyzed by a one-way ANOVA. A value of *p* < 0.05 was considered statistically significant.

## 4. Conclusions

In summary, research on antioxidants, especially exploration of natural compounds with low cytotoxicity, has become an important area of biomedicine. *cis*-*Z,Z'*-3a.7a',7a.3a'-Dihydroxyligustilide is a new compound isolated directly from AS that showed antioxidant activity on HCAECs. This compound also reduce the apoptosis of HUVEC, and enhance HUVEC proliferation *in vitro*. These results demonstrated that *cis*-*Z,Z'*-3a.7a',7a.3a'-dihydroxyligustilide may be a potential anti-oxidant agent which had a protective effect against HUVEC injuries induced by H_2_O_2_ *in vitro*. Its anti-oxidative activity could effectively relieve the H_2_O_2_-induced cell cycle arrest in HUVECs beneficial to the resumption of cell proliferation. These findings may also suggest that decreased cell death can be related to the ROS-scavenging capacity of compound **1**. Further studies need to be carried out in order to elucidate these mechanisms in HCAECs.
